# Finite Model Analysis and Practical Design Equations of Circular Concrete-Filled Steel Tube Columns Subjected to Compression-Torsion Load

**DOI:** 10.3390/ma14195564

**Published:** 2021-09-25

**Authors:** Yongzhi Gong, Faxing Ding, Liping Wang, Borong Huang, Yingjie Shan, Fei Lyu

**Affiliations:** 1 School of Civil Engineering, Central South University, Changsha 410075, China; gyzcsu@csu.edu.cn (Y.G.); dinfaxin@csu.edu.cn (F.D.); wlp2016@csu.edu.cn (L.W.); huangbr07@outlook.com (B.H.); syjcsu@csu.edu.cn (Y.S.); 2 Engineering Technology Research Center for Prefabricated Construction Industrialization of Hunan Province, Changsha 410075, China

**Keywords:** concrete-filled steel tubes, compression-torsion, composite action, finite element, ultimate strength, confinement effect

## Abstract

The objective of this study is to investigate the mechanical properties and the composite action of circular concrete-filled steel tube (CFST) columns subjected to compression-torsion load using finite element model analysis. Load–strain (T–γ) curves, normal stress, shear stress, and the composite action between the steel tubes and the interior concrete were analyzed based on the verified 3D finite element models. The results indicate that with the increase of axial force, the maximum shear stress at the core concrete increased significantly, and the maximum shear stress of the steel tubes gradually decreased. Meanwhile, the torsional bearing capacity of the column increased at first and then decreased. The torque share in the columns changed from the tube-sharing domain to the concrete-sharing domain, while the axial force of the steel tube remained unchanged. Practical design equations for the torsional capacity of axially loaded circular CFST columns were proposed based on the parametric analysis. The accuracy and validity of the proposed equations were verified against the collected experimental results.

## 1. Introduction

Concrete-filled steel tube (CFST) structures are widely used as compression members in bridges and building structures. Compared with conventional reinforced concrete columns, concrete-filled steel tube columns have the advantages of making full use of the material strength of steel and concrete, high bearing capacity and rigidity, and excellent seismic performance [1,2,3,4,5]. Hence, the performance and mechanical properties of CFST columns under axial loading have received a great deal of attention [6,7,8,9,10,11,12]. There have been many experiments, theoretical models, finite element models, and design methods based on the compression-bending and tensile-bending performance of CFST columns [13]. However, in projects such as curved bridges, offshore oil platforms, and other complex structures, the CFST columns are subjected to a combined compression-torsion loading state [12,14,15]. Thus, a thorough understanding of the mechanical properties of CFST columns under axial compression-torque is essential.

Extensive studies have been carried out on CFST columns subjected to axial compression [16,17,18,19] and compression-bending loads [20,21,22]. In addition, some scholars have started experimental and numerical explorations into the compression-torsion behaviors of circular CFST columns. Through experiments and finite element analysis, Han [23] defined the torsional bearing capacity index of CFST tube pure torsion members and gave a simplified calculation formula for torsional bearing capacity. Beck et al. [24], Han et al. [25], Eun-Taik et al. [26], and Ding et al. [27] have conducted experimental and numerical analyses of the behavior of circular CFST columns under pure torsion and proposed corresponding calculation methods and calculation formulas. A theoretical model making use of layered cylinders for the complete process analysis of CFST columns under combined compression-torsion forces was proposed by Nie et al. [28]. 

Xu et al. [29] performed a series of compression-torsion tests on circular CFST stubs with two different steel rate designs (steel rate as low as 6.4%, and as high as 17.8%). As the axial compression ratio increased, the torsional capacity of the CFST column first increased and then decreased. In addition, they proposed that the cracking and deformation of concrete in compression-torsion conditions still follow the “spiral effect” pattern. Jin et al. [30] conducted a simplified calculation of the entire torsion process of CFST columns based on experimental research on the torsion performance of concrete-filled steel tube members and existing standard calculation methods. Nie et al. [14] carried out a quasi-static test on eight CFST columns and found that the hysteresis curves of CFST columns under pure torsion and low compression–torsion cyclic load were plump. The monotonic pure torsion loading, reciprocating pure torsion loading, and eccentric reciprocating torsion pseudo-static tests of specimens of different heights of square steel tube and CFST columns were completed by Wang et al. [15]. The torque–torsion angle hysteresis relationship, steel pipe strain change law, damage mode, and failure mechanism are discussed. Wang et al. [31] conducted experimental research on concrete-filled steel tube columns under pure torsion, bending-shear, and combined bending-shear–torsion; they then compared and analyzed the mechanical properties and working mechanism of specimens under different loads and provided a formula for calculating the bearing capacity of columns under complex bending, shear, and torsion.

In terms of theoretical approaches, Lee et al. [32] established a finite element nonlinear analysis method for the torsion force of CFST column members based on the torsion testing of CFST tube single-circular tube members, using finite element solid element modelling. Sudip et al. [33] studied the effect of the slenderness ratio on the torsional behavior of CFST columns. Wang et al. [34] studied the torsional effect of circular CFST columns in curved beam bridges. A laminated tubes model for analyzing the mechanical behavior of CFST columns under combined axial force–torsion action was presented and then used to obtain the entire loading history of CFST columns. Abovementioned studies have revealed that when the axial force is small, the increase in axial force can improve the torsional capacity of CFST columns. As the axial force is increased, it tends to weaken torsional resistance. These findings can help provide an understanding of the compression-torsion behavior of CFST columns. However, there are still many issues that need further investigation, including the influencing mechanism of the composite action between steel and concrete on the bearing capacity of CFST columns. In addition, the analyses of stress state and torque distribution between the steel tubes and the interior concrete are still limited.

In recent years, Ding et al. [27] put forward a triaxial plastic-damage constitutive concrete model and applied it into the pure torsion simulation of CFST columns. The finite element model could effectively calculate the stress–strain state of steel tubes and concrete. The steel–concrete composite effect on the pure torsional bearing mechanism was also obtained; however, its applicability to the simulation of complex loading conditions has not been verified. Since the current study is based on the proposed finite element modelling method, a further investigation of the compression-torsion behavior of circular CFST columns was performed. By using the triaxial plastic-damage concrete model, the circular CFST column subjected to compression-torsion was established, and the shear stress state of the encased steel tube and the concrete were analyzed. The influence of various construction details on compression-torsion properties was discussed using a parametric study, and calculation formulas for the practical torsion capacity of circular CFST columns under different axial pressure ratios were also developed. The proposed formulas can take the contribution of axial force into account, and they possess high accuracy and simple form.

This paper is organized as follows: In Section 1, the background and motivation are expressed; in Section 2, the finite element model used in this study is introduced and its validity is examined; and in Section 3, the effect of the loading path is studied. In addition, the composite action and load sharing ratio of the steel tubes and the core concrete are analyzed. Section 4 presents a parametric study to determine the influence of various parameters on the bearing capacity of the composite columns. Finally, Section 5 concludes the paper by presenting the major findings of the study.

## 2. Finite Element Modelling of Circular CFST Columns

### 2.1. Finite Element Models

**Figure 1:**
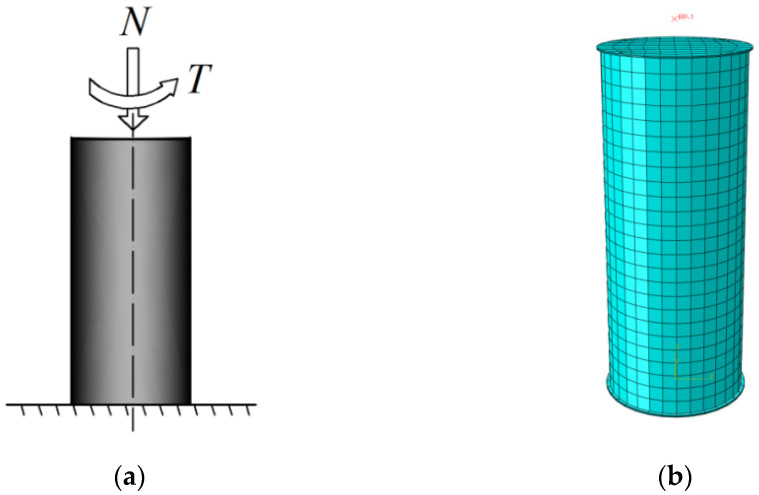
FE model of CFST stub column: (**a**) Loading path of CFST columns; (**b**) mesh result.

The finite element (FE) models of circular CFST columns were established using ABAQUS version 6.14. The adopted plastic-damage constitutive model of concrete and the elastoplastic hardening model of steel can be found in Ding et al. [27]. In the FE model, the outer steel tube and infilled concrete are simulated by the solid element C3D8R, and the upper and bottom loading plates are modelled by the rigid body element. The interface action between the loading plate and the column, and between the inner surface of steel tube and the outer surface of the concrete, are simulated by the contact surface model. Other details, such as element meshing and boundary conditions, are the same as in the reference work by Ding et al. [27]. The established FE model of the circular CFST column is shown in Figure 1.

### 2.2. Validation of FE Models

**Figure 2:**
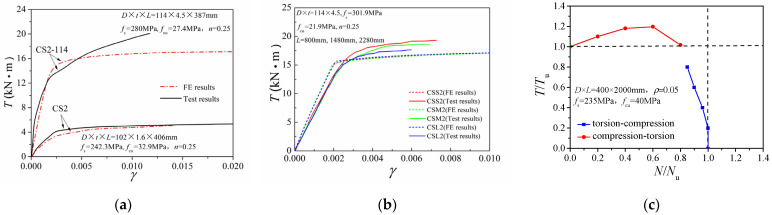
Comparison between the *T*–*γ* curve results of the FE model and the test: (**a**) CS2 and CS2-114; (**b**) CSS2, CSM2 and CSL2; (**c**) C2-1.

FE simulations of several related compression-torsion tests were conducted on the circular CFST columns, and the torsional moment comparisons with their corresponding test data are presented in Table 1. Here *D* is the column diameter, *t* represents the panel thickness of the steel tube, *f*_s_ is the yield strength of the steel, *f*_cu_ is the compressive strength of the concrete, *N* is the applied axial force, and *n* indicates the axial pressure ratio. The average ratio of test torsional moment (*T*_u,1_) to simulation results (*T*_u,0_) was 1.085. The corresponding variation coefficient was 0.106, indicating the efficacy of this FE model it calculating the torsional capacity of the circular CFST column. Figure 2 compares the torsional moment versus shear strain (*T*–*γ*) curves obtained from the two approaches. The results show good agreement between test data and the finite element curve in the elastic section. Therefore, this FE model was applied to the investigation of compression-torsion properties.

**Figure 3:**
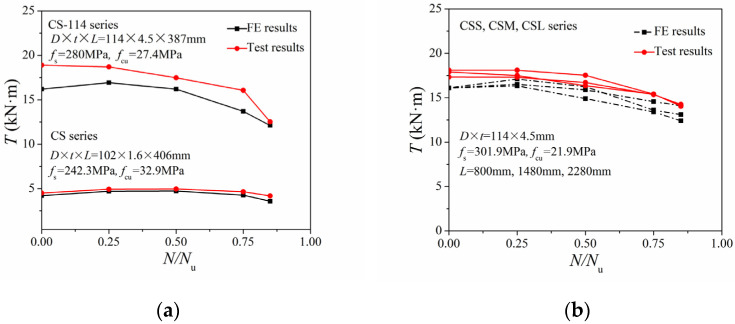
Comparison of FE results and test results of *N*/*N*_u_~*T*/*T*_u_ correlation curves: (**a**) CS and CS-114 series; (**b**) CSS, CSM, and CSL series.

Figure 3 displays the comparison of the *N*/*N*_u_~*T*/*T*_u_ correlation curves from the FE results and test data. The FE results were slightly higher than the corresponding experimental values, but they followed similarly varying trends. For the specimens with a low steel ratio (C.S. series, *ρ* = 4.5%), as the axial compression ratio increased, the torsional bearing capacity increased at first and then decreased. Conversely, for specimens with a high steel ratio (CSS, CSM, and CSL series, *ρ* = 17.8%), the torsion strength decreased slowly, with an increase in the axial compression ratio. Since in practical projects the steel ratio of CFST columns is generally low (*ρ* < 10%), investigations on low-steel-ratio cases are critical. Hence, only the low steel ratio specimens we subject to further investigations in terms of compression-torsion properties.

## 3. Investigation of CFST Columns Subjected Compression-Torsion

### 3.1. Effect of Loading Path

In compression-torsion problems, the applied axial load and torque are used as independent variables, then the loading process can be subdivided with different sequences, and the effects of different loading paths need to be identified first. Given a typical circular CFST column constructed with a Q235 circular steel tube and C40 concrete with a steel ratio of 0.05, a section diameter D of 400 mm, and a column length L of 2000 mm, the ultimate axial bearing strength could be obtained through Equation (1) [27]:

*N*_u_ = *f*_c_*A*_c_ (1 + 1.7*φ*) (1)

Here *φ* is the confinement index coefficient, *φ* = *A*_s_*f*_s_/*A*_c_*f*_c_. The pure torsional strength of these circular CFST columns can also be obtained with Equation (2), proposed by Ding et al. [27]

*T*_u_ = 1.15*A*_so_*t*_s_
*f*_s_ + 0.67*A*_c_*r*_c_*τ*_cc_ (2)

where *A*_so_ is the area bounded by the centerline circle of the steel tube, calculated as *π*(*r*_c_ + *t*/*2*)*^2^*. *A*_c_ is the core concrete area, *f*_c_ is the compressive strength of concrete, *r*_c_ is the section radius of the core concrete, and *τ*_cc_ is the maximum shear stress of the core concrete and equals (0.37 + 6.0*ρ*) × *f*_cu_^0.62^. In the equations presented in this paper, the units of the area and the material strength are mm^2^ and MPa, respectively.

**Figure 4:**
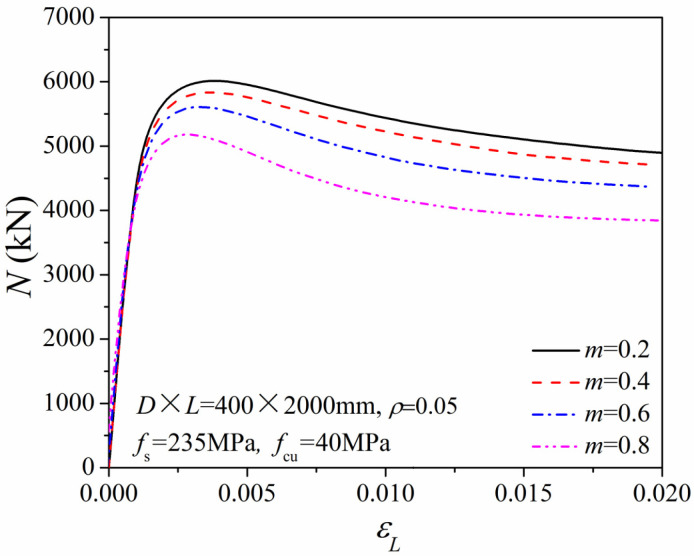
*N*–*ε*_L_ relationship of the compression-torsion columns.

**Figure 5:**
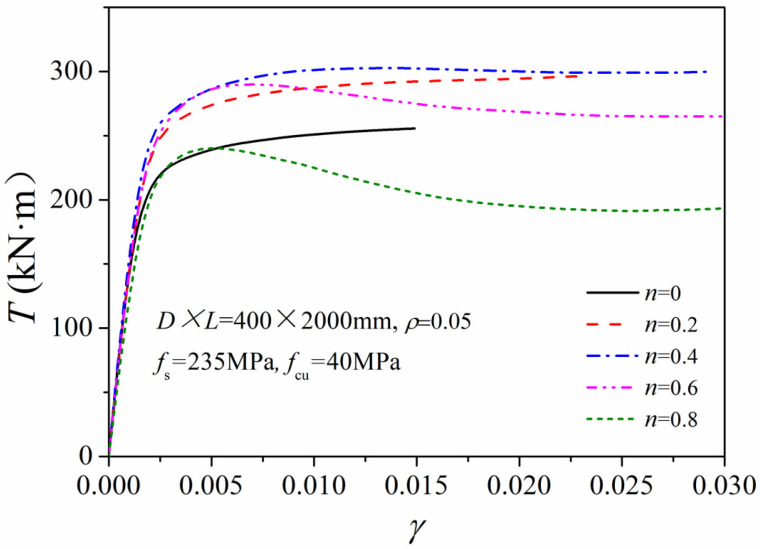
*T*–*γ* relationship of the compression-torsion columns.

With the axial capacity *N*_u_ and torsional strength *T*_u_, we can realize different torque ratios (*m* = *T*/*T*_u_) and axial pressure ratios (*n* = *N*/*N*_u_) in numerical simulations by changing the applied axial and the torsional forces. Figure 4 shows the relationship between axial force *N* and the average longitudinal strain *ε*_L_ of the columns under different torque ratios (*m* = *T*/*T*_u_). With different axial pressure ratios, the initial stiffness remained almost constant. Moreover, as the applied torque *T* gradually increased, the torsional capacity continued to decrease. Figure 5 gives the relationship of torsional strength T to averaged shear strain *γ* (*T*–*γ* curve) under different axial pressure ratios (*n* = *N*/*N*_u_). The shear stiffness of the column under combined compression-torsion conditions was nearly the same as that under pure torsion conditions. In addition, with an increase in the axial compression ratio, the ultimate torsional strength first increased and then decreased.

**Figure 6:**
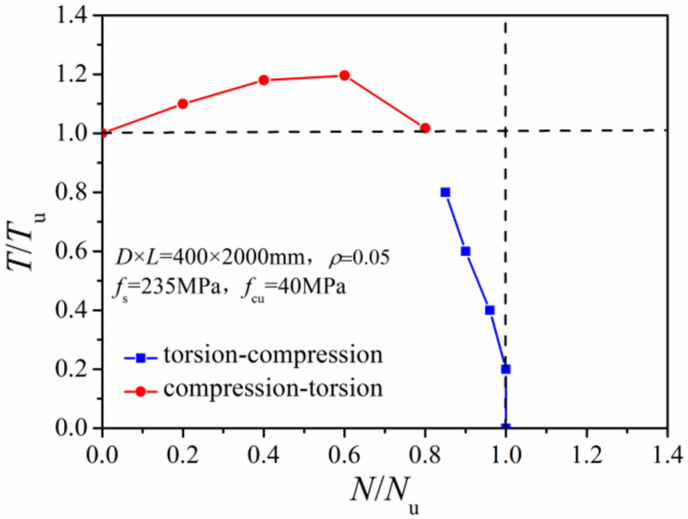
Comparison of *N/N_u_~T/T_u_* relationships under different loading paths.

Figure 6 gives the *N*/*N*_u_~*T*/*T*_u_ correlation curves of a typical circular CFST column under the above two loading paths. The torsion-first loading case weakened the axial-force-resisting ability of the CFST column. In contrast, the compression-first loading path exerted a strengthening effect on the torsional strength. When the axial pressure ratio varied between 0.2 and 0.6, the torsional bearing capacity increased by 10–20%. As the ratio of axial force to compression strength increased to 0.8, the torsion resistance decreased slightly, but the torsional capacity remained nearly equivalent to that of the pure torsional column.

### 3.2. Composite Action and Load Sharing Ratio

**Figure 7:**
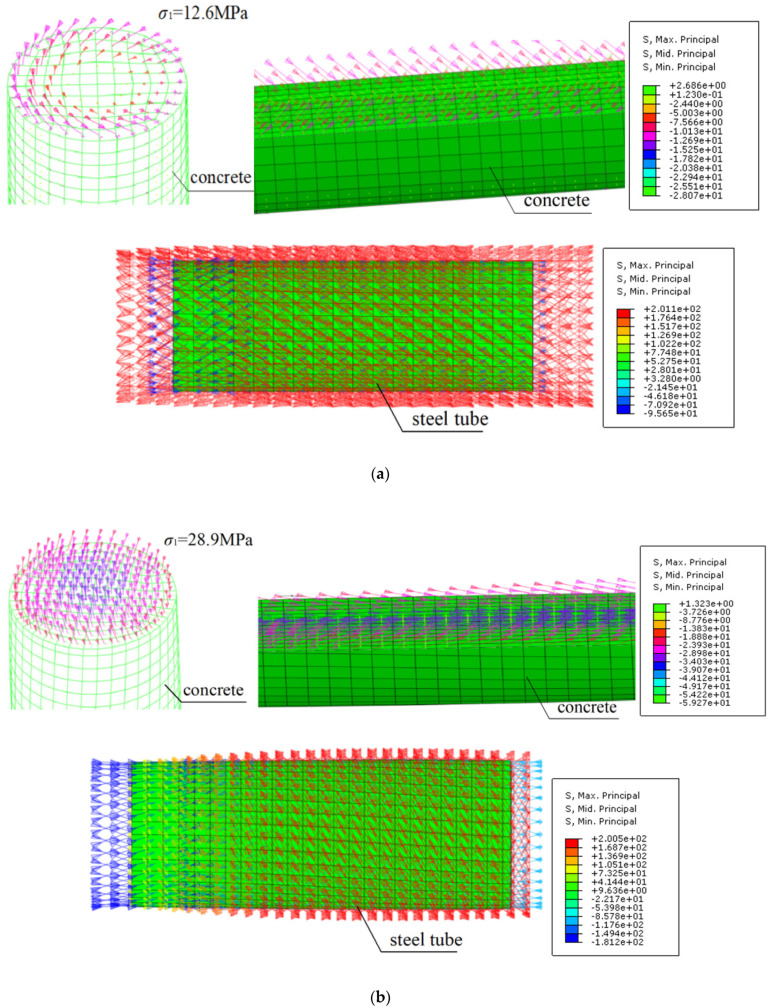
Stress state of the concrete and steel tube under pure torsion and compression-torsion: (**a**) *n* = 0; (**b**) *n* = 0.6.

Figure 7 gives the principal stress distribution of the core concrete and encased steel tube of the calculated CFST column under a compression-torsion loading state. Under the pure torsion state (*n* = 0), compression stress was induced at the core concrete. The maximum principal stress of the concrete was 12.6 MPa. Moreover, the internal stress followed a downward helical pattern, with the angle between the principal stress direction and column axis being smaller than 45°. At the same time, tension stress was induced in the steel tubes to ensure the forces’ equilibrium state. Thus, the mechanics of the circular CFST column in the pure torsion state is a combination of “pulling torsion” at the steel tube and “compressive torsion” at the interior concrete. For the *n* = 0.6 loading case, the angle between the concrete compressive stress and the column axis was about 30°. Compared to the pure torsion column, the internal compressive stress at the interior concrete was more uniform across the section, with the maximum principal stress reaching 28.9 MPa. Therefore, the internal stress state of the CFST column under compression-torsion state can be simplified as a combination of the “pulling (compressive) torsion” of the steel tube and the “compressive torsion” of the concrete. The stress state of the steel tube depended on the axial force condition. When axial force was low with an increase of applied torsional moment, the internal stress in the steel tube changed from compression to pulling. When the axial force level was high, the steel tube was always in a compression state.

**Figure 8:**
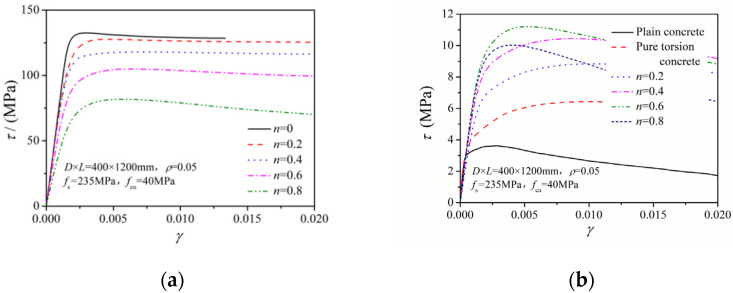
*τ–γ* relation of the steel tube and concrete under different axial compression ratios: (**a**) Steel tube; (**b**) core concrete.

Figure 8 compares the maximum shear-stress–shear-strain relation at the steel tube and the core concrete under different axial compression ratios. The maximum shear stress of the encased steel tube gradually reduced with increasing axial pressure ratio. The extent of stress reduction was small in low compression ratio cases and large under high compression ratio conditions. With increasing axial pressure ratio, the maximum shear stress of the core concrete increased remarkably at first and then gradually decreased. In addition, the maximum shear stress at the concrete increased by 35–80% compared to the maximum shear stress of the corresponding pure torsion column.

**Figure 9:**
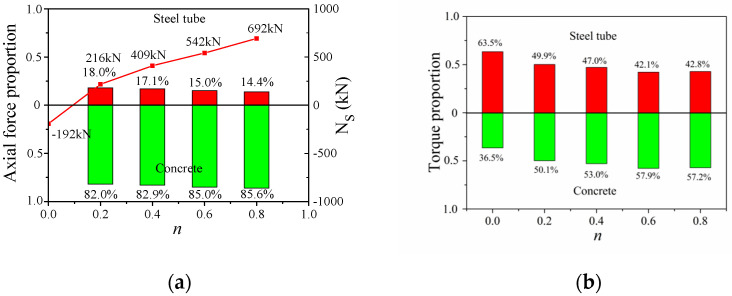
Load-bearing proportion of steel tube and concrete: (**a**) Axial force proportion and *N_s_*; (**b**) torque proportion.

Figure 9 shows the axial force and torque-sharing proportion between the steel tubes and the interior concrete at the ultimate loading state. When the CFST column was under the ultimate loading state, the applied axial force was resisted by the interior concrete with about an 85% sharing ratio, as shown in Figure 9a. The steel tubes were under a tension state in the pure torsion column, and the actual axial force was negative. With an increase in the axial compression ratio, the actual axial force resisted by the steel tubes gradually increased, and the axial-load-sharing proportion of the steel tubes reduced from 18% to 14.4%. In Figure 9b, the torque shared by the steel tubes was 63.5% for the pure torsion specimens. With increasing axial pressure ratio, the maximum shear stress at the steel tubes gradually decreased, and the torque sharing proportion gradually decreases to about 42%. The torque sharing changed from the tube-sharing domain to the concrete-sharing domain.

**Figure 10:**
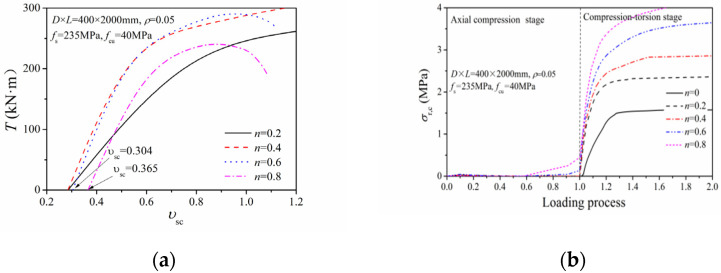
Composite action of steel tube and concrete: (**a**) Relation of load–strain ratio; (**b**) lateral compressive stress of concrete.

Figure 10 displays the relation of torque *T* to stain ratio *υ*_sc_ and the lateral compressive stress (*σ*_r,c_) of concrete. For axial pressure ratios of 0.2 and 0.4, the initial stain ratio was 0.285, which is close to Poisson’s ratio of steel. Therefore, the steel tubes presented little constraining effect on the interior concrete during the axial loading stage, and the core concrete had no lateral compressive stress. When the axial pressure ratios were 0.6 and 0.8, the initial stain ratios were 0.304 and 0.365, respectively, together with an increased lateral compression stress on the concrete. This indicates that when the axial pressure was relatively large, the steel tubes had a solid clamping effect on the core concrete, resulting in a three-axis compression state affecting the interior concrete. Compared to the pure-torsion column, the lateral compressive stress on the core concrete increased dramatically in the compression-torsion column, together with the restraining effect from the steel tubes on the interior concrete. Therefore, the torsional resistance of the compression-torsion column was generally more prominent than that of the pure-torsion column.

In summary, the mechanics of a circular CFST column under compression-torsion can be regarded as a combination of “pulling (compressive) torsion” at the steel tube and “compressive torsion” at the concrete. Due to the composite effect of the steel tube and the concrete under axial force, the maximum shear stress at the core concrete increased significantly while shear stress at the steel tube gradually reduced. Therefore, as the axial compression ratio increased, the torsion capacity of the compression-torsion column initially increased and then gradually decreased.

## 4. Practical Design Equations

### 4.1. Parametric Study

#### 4.1.1. Material Strength

**Figure 11:**
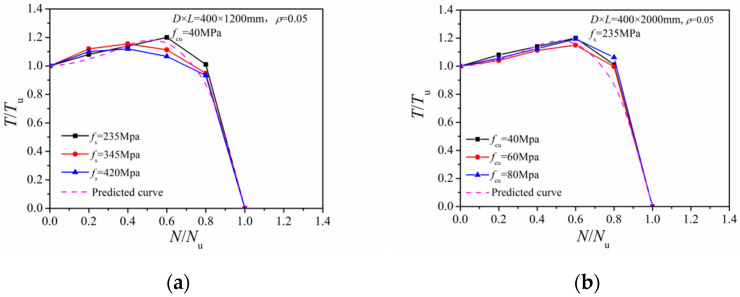
Effect of different steel and concrete strengths: (**a**) Steel yield strength; (**b**) concrete strength.

Figure 11 compares *N/N_u_~T/T_u_* correlation curves with different steel yield and concrete strength. Only minor differences are present between the different curves, which indicates that the strength of the steel and the concrete exert little influence on the ultimate torsional capacity of the CFST column.

#### 4.1.2. Steel Ratio

**Figure 12:**
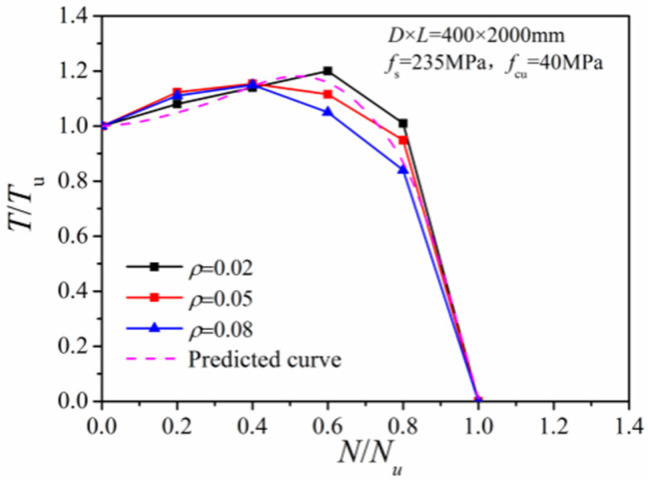
Effect of different steel ratios.

Figure 12 gives comparisons of the *N/N_u_~T/T_u_* correlation curves with different steel ratios. When the axial compression ratio was between 0.2 and 0.4, there was nearly no difference between the curves obtained with different steel ratios. When the axial pressure increased to 0.6–0.8, a greater steel ratio induced a larger torque share at the steel tube. The case with a greater axial force ratio had a more significant shear stress reduction at the steel tube and a reduced *T/T_u_* ratio.

#### 4.1.3. Slenderness Ratio

**Figure 13:**
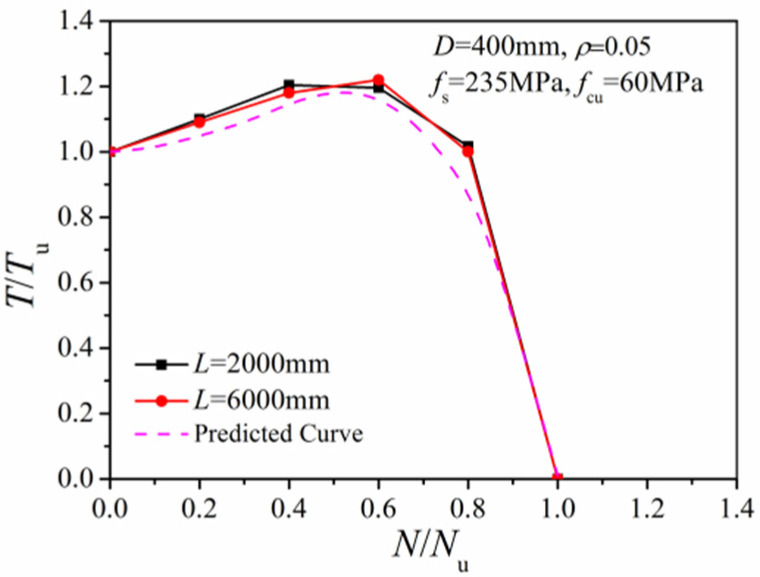
Effect of different slenderness ratios.

Figure 13 compares the *N/N_u_~T/T_u_* correlation curves with 2000 mm and 6000 mm column length (corresponding to slenderness ratios of 5 and 15, respectively). Results indicate little influence of the slenderness ratio on the torsional bearing capacity of the column.

#### 4.1.4. Column Dimension

**Figure 14:**
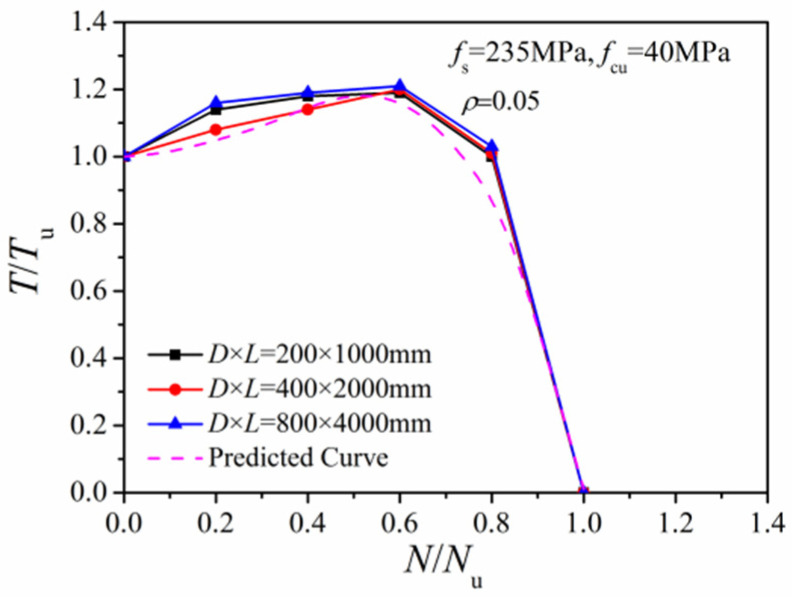
Effect of different column sizes.

Figure 14 presents comparisons of the *N/N_u_~T/T_u_* correlation curves with different column dimensions. The curves under different column dimensions are nearly coincident, indicating a small effect of column size on torsional strength.

### 4.2. Simplified Formula of Torsion Capacity

**Figure 15:**
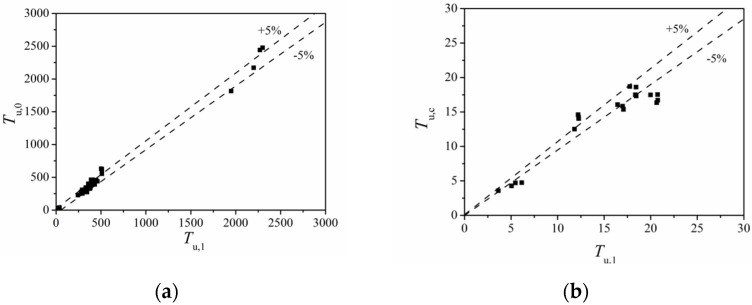
Calculation formula verification result: (**a**) FE values and formula values; (**b**) test and formula values.

Parameters like material strength, steel ratio, slenderness ratio, and column dimensions exerted little influence on the *N/N_u_~T/T_u_* curve of the compression-torsion column. Through the 36 FE *N/N_u_~T/T_u_* calculation results in Figure 15, the relation of Equation (3) can be calibrated as:

(3) $$\frac{T}{T_{u}}=-4.5{\left(\frac{N}{N_{u}}\right)}^{3}+4{\left(\frac{N}{N_{u}}\right)}^{2}-0.5(\frac{N}{N_{u}})+1$$

where *T*_u_ and *N*_u_ can be calculated according to Equations (1) and (2), respectively. To improve the practicability of Equation (3), it can be further simplified in favor of safety. The torsional ultimate bearing capacity of the circular CFST column at different axial compression ratios can be simplified as:

(4) $$\frac{T}{T_{u}}=\left\{ \begin{array}{l} 1\;n\leq 0.8\\ 1-\frac{N}{N_{u}}0.8<n\leq 1 \end{array}\right.$$

### 4.3. Formula Validation

Figure 15 gives the comparison of 36 FE values (*T*_u,0_) and 20 test values (*T*_u,c_) with corresponding calculated values (*T*_u,1_) using Equation (3). The average value of *T*_u,1_/*T*_u,0_ ratio was 1.001, and the variation coefficient was 0.093. The average value of *T*_u,c_/*N*_u,1_ ratio was 1.001, and the variation coefficient was 0.132. The comparisons verify the effectiveness and accuracy of the proposed formulas.

**Figure 16:**
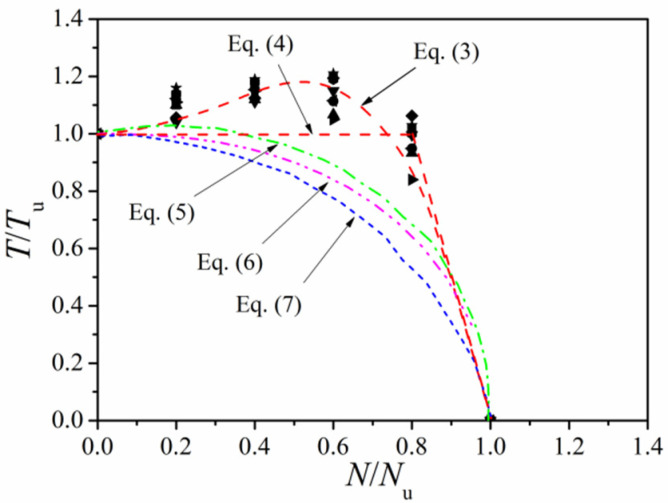
Comparison of design equations.

Table 2 lists the formulas for the torsional capacity of compression-torsion circular CFST columns found in the existing literature. Figure 16 shows the comparison of *N/N_u_~T/T_u_* correlation curves calculated with each formula. In addition, Table 3 displays the comparisons of test values (*T_u,c_*), FE values (*T_u,0_*), and the calculated values (*T_u,1_*) together with the proposed formula and other formulas in Table 2. It indicates that the calculated strength of the proposed formula fits the experimental results with better accuracy and less dispersion.

### 4.4. Composite Shear Stiffness Formula

According to Ding et al. [27], the composite shear stiffness of a pure torsion circular CFST column needs to consider the weakening effect of plastic deformation at the constrained core concrete. Therefore, the composite shear stiffness can be expressed as:

*G*_sc_*A*_sc_ = *G*_s_*A*_s_ + *k*_t_*G*_c_*A*_c_ (8)

where *G_sc_A_sc_* is the composite shear stiffness and *G_sc_* is the composite shear modulus. *G_s_* and *G_c_* are the shear moduli of the steel and the concrete, *A_sc_* is the section area, and *A_s_* and *A_c_* are the areas of the steel tube and the concrete, respectively.

**Figure 17:**
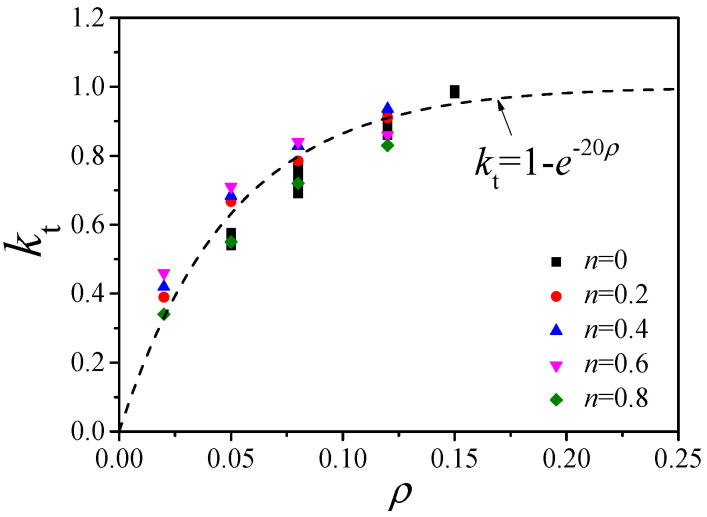
*k_t_–ρ* correlation curve of the compression-torsion columns.

Figure 17 compares the composite shear stiffness of compression-torsion columns from FE simulations and formula calculations with Equation (8). The *k_t_* values of the compression-torsion columns with different axial compression ratios were still in agreement with the fitted curve, resulting in a minor effect of the axial compression ratio on the composite shear stiffness of circular CFST columns.

## 5. Conclusions

In this study, systematic FE analyses of circular CFST columns subjected to axial compression-torsion were carried out. The main conclusions are as follows:

- The loading path can influence the compression or torsion strength performance. Torsion-first loading can weaken the axial-force-resisting ability of the CFST column, while the compression-first loading path has an enhancing effect on the torsional strength.
- Due to the composite action of the steel tubes and the concrete, the maximum shear stress is significantly increased at the core concrete and gradually reduced at the steel tubes. As the axial pressure ratio increases, the maximum shear stress at the core concrete increases at first and then decreases.
- As the axial pressure ratio increases, the axial load share of the steel tubes remains nearly unchanged, and the maximum shear stress share gradually decreases. The torque sharing in the circular CFST column changes from the tube-sharing domain to the concrete-sharing domain.
- Formulas for the torsion capacity of circular CFST columns are presented based on parametric analysis. The formulas can account for the contribution of axial force in a highly accurate yet simple form.

## Figures and Tables

**Table 1:** Comparison between the test and FE results.

Specimen	*D* × *t* × *L* (mm)	*f*_s_ (MPa)	*f*_cu_ (MPa)	*n*	*T*_u,1_	*T*_u,0_	*T*_u,1_/*T*_u,0_	Ref.
					(kN m)	(kN m)		
CS2	102.4 × 1.6 × 406	242.3	32.9	0.25	4.7	4.93	0.953	[29]
CS3				0.5	4.73	4.94	0.957	
CS4				0.75	4.26	4.65	0.916	
CS5				0.85	3.57	4.18	0.854	
CS2-114	114 × 4.5 × 387	280	27.4	0.25	18.7	16.92	1.105	[29]
CS114				0.5	17.47	15.68	1.114	
CS4-114				0.75	16.06	13.62	1.179	
CS5-114				0.85	12.51	11.62	1.077	
CSS2	114 × 4.5 × 800	301.9	21.9	0.25	18.61	17.1	1.088	[35]
CSS3				0.5	17.53	16.24	1.079	
CSS4				0.75	15.41	13.6	1.133	
CSS5				0.85	14.05	11.31	1.242	
CSM2	114 × 4.5 × 1480	301.9	20.9	0.25	17.5	15.34	1.141	
CSM3				0.5	16.35	14.64	1.117	
CSM4				0.75	15.84	12.46	1.271	
CSM5				0.85	14.6	10.89	1.341	
CSL2	114 × 4.5 × 2280	301.9	21.9	0.25	17.31	16.52	1.048	
CSL3				0.5	16.71	15.83	1.056	
CSL4				0.75	15.36	14.54	1.056	
CSL5				0.85	14.25	14.1	1.011	
C2-1	220 × 6 × 1100	336	49.4	0.2	125.1	117.6	1.064	[14]
Average value							1.085	
CV							0.106	

**Table 2:** The ultimate bearing capacity calculation formulae in the existing literature.

No.	Equations		References
1	$(\frac{N}{\phi A_{\mathrm{sc}}f_{\mathrm{sc}}}{)}^{3}+(\frac{T}{\gamma w_{\mathrm{sc}}{}^{T}\tau_{\mathrm{sc}}{}^{y}}{)}^{2}=1$	(5)	[23]
2	$T_{\mathrm{cr}}=1.25(T_{c}+T_{s})$ $T_{c}=\frac{2}{3}\pi r^{3}f_{s}\sqrt{1+\frac{\sigma_{c}}{f_{c}}}(1-0.98{\left(\frac{\sigma_{c}}{f_{c}}\right)}^{4.5})$ $T_{c}=\frac{2}{3}\pi (r_{1}^{3}-r_{0}^{3})\sqrt{1-{\left(\frac{P}{P_{y}}\right)}^{2}}\frac{f_{y}}{\sqrt{3}}$	(6)	[29]
3	$(\frac{T}{T_{u}}{)}^{2}=1-\frac{N}{N_{cu}}$	(7)	[34]

**Table 3:** Comparison of different calculation formulas.

Specimen	FE	Equation (3)	Equation (5)	Equation (6)	Equation (7)	Ref.
CS2	0.953	0.861	0.915	0.925	1.089	[29]
CS3	0.957	0.769	0.977	1.015	0.953	
CS4	0.916	0.843	1.082	1.165	0.965	
CS5	0.854	0.983	1.11	1.213	0.874	
CS2-114	1.105	1.053	1.119	1.131	1.045	[29]
CS114	1.114	0.874	1.109	1.152	1.01	
CS4-114	1.179	0.977	1.254	1.35	0.963	
CS5-114	1.077	1.059	1.196	1.307	0.992	
CSS2	1.088	1.010	1.074	1.085	0.995	[35]
CSS3	1.079	0.845	1.073	1.114	0.994	
CSS4	1.133	0.903	1.16	1.249	0.903	
CSS5	1.242	1.146	1.294	1.415	0.851	
CSM2	1.141	0.954	1.014	1.025	1.073	
CSM3	1.117	0.792	1.005	1.044	1.08	
CSM4	1.271	0.933	1.198	1.29	0.964	
CSM5	1.341	1.197	1.352	1.477	0.874	
CSL2	1.048	0.939	0.999	1.009	1.06	
CSL3	1.056	0.805	1.022	1.062	1.031	
CSL4	1.056	0.900	1.156	1.245	0.946	
CSL5	1.011	1.163	1.313	1.435	0.745	
C2-1	1.064	1.116	1.148	1.155	0.896	[14]
Average	1.085	0.958	1.122	1.184	0.967	
CV	0.106	0.132	0.104	0.129	0.090	

## Data Availability

No new data were created or analyzed in this study. Data sharing is not applicable to this article.
